# Methodological challenges and solution strategies during implementation of a midwife-led multicenter randomized controlled trial (RCT) in maternity hospitals

**DOI:** 10.1186/s12874-021-01429-0

**Published:** 2021-10-25

**Authors:** Sabine Striebich, Elke Mattern, Theresa Oganowski, Rainhild Schäfers, Gertrud Ayerle

**Affiliations:** 1 Martin Luther University Halle-Wittenberg, Institute of Health and Nursing Science, Magdeburger Str. 8, 06112 Halle (Saale), Germany; 2 Hochschule für Gesundheit Bochum - University of Applied Sciences, Gesundheitscampus 6 – 8, 44801 Bochum, Germany

## Abstract

**Background:**

Randomized controlled trials (RCTs), especially multicentric, with complex interventions are methodically challenging. Careful planning under everyday conditions in compliance with the relevant international quality standard (Good Clinical Practice [GCP] guideline) is crucial. Specific challenges exist for RCTs conducted in delivery rooms due to various factors that cannot be planned beforehand. Few published RCTs report challenges and problems in implementing complex interventions in maternity wards. In Germany as well as in other countries, midwives and obstetricians have frequently little experience as investigators in clinical trials.

**Methods:**

The aim is to describe the key methodological and organizational challenges in conducting a multicenter study in maternity wards and the solution strategies applied to them. In particular, project-related and process-oriented challenges for hospital staff are considered. The exemplarily presented randomized controlled trial “BE-UP” investigates the effectiveness of an alternative design of a birthing room on the rate of vaginal births and women-specific outcomes.

**Results:**

The results are presented in five sectors:

1) Selection of and support for cooperating hospitals: they are to be selected according to predefined criteria, and strategies to offer continuous support in trial implementation must be mapped out.

2) Establishing a process of requesting informed consent: a quality-assured process to inform pregnant women early on must be feasible and effective.

3) Individual digital real-time randomization: In addition to instructing maternity teams, appropriate measures for technical failure must be provided.

4) The standardized birthing room: The complex intervention is to be implemented according to the study protocol yet adapted to the prevailing conditions in the delivery rooms.

5) GCP-compliant documentation: midwives and obstetricians will be instructed in high-quality data collection, supported by external monitoring throughout the trial.

**Conclusion:**

Since not all potential challenges can be anticipated in the planning of a trial, study teams need to be flexible and react promptly to any problems that threaten recruitment or the implementation of the complex intervention. Thought should be given to the perspectives of midwives and obstetricians as recruiters and how clinic-intern processes could be adapted to correspond with the trial’s requirements.

**Trial registration:**

The BE-UP study was registered on 07/03/ 2018 in the German Register for Clinical Trials under Reference No. DRKS00012854 and can also be found on the International Clinical Trials Registry Platform (ICTRP) (see https://apps.who.int/trialsearch/Trial2.aspx?TrialID=DRKS0001285).

## Background

### Quality of RCTs

Randomized controlled trials (RCTs) are the gold standard in clinical research; they generate findings with the highest level of evidence for improving the quality of patient care. RCTs are methodically complex and costly, and their implementation brings special challenges and hindrances [1]. Using a complex intervention is particularly demanding; the new Medical Research Council guidance specifies how a complex intervention should be developed, established and reproduced [2], and there are also special requirements regarding the quality of the reporting of such studies [3].

Careful and pragmatic planning under everyday conditions in compliance with the relevant international quality standard (Good Clinical Practice [GCP] guideline [4]) is crucial for the quality of an RCT. Continuous monitoring in the study centers serves on the one hand to guarantee the rights of the study participants, for example, checking that ethically sound informed consent is carried out and written informed consent is obtained. On the other hand, the quality of the data must be guaranteed through randomization in line with the study protocol, careful and valid data collection, recording adverse events [5], and a follow-up that is as complete as possible. Unforeseen challenges must be anticipated when implementing RCTs [6], meaning that problems in the course of the study must be recognized in good time and that appropriate solutions must be continuously developed, applied and re-evaluated. In this vein, it is helpful to involve user representatives to ensure that the perspective and needs of service-users are taken into account as far as possible [7]. Furthermore, when applying a complex intervention, it is important to evaluate the process to identify hindrances and challenges that might exist regarding the implementation of the study under everyday conditions, the practicability of the intervention and its acceptance by staff and patients [2].

### Well known problems in the implementation of RCTs

When planning RCTs, especially multicenter RCTs, it is frequently underestimated that the actual number of recruited study participants can often be distinctly lower than the number calculated as the monthly recruiting rate. Two reviews report that only 31 to 55% of trials recruited their originally specified target sample size [8, 9]. Specific challenges exist for RCTs conducted in delivery rooms: large teams of midwives and obstetricians work together, which means that there is not just one “principal investigator” conducting the study intervention but that the whole team in the obstetric unit is involved in implementing the study. Moreover, women arrive in the delivery room 24 h a day, seven days a week, and this can result regularly in “peak hours” with bed shortages and a high work burden for the whole team.

Due to heterogeneous healthcare systems, different conditions exist internationally regarding the spectrum of tasks, the cooperation between midwives and obstetricians, and the procedures within the actual birthing room. Furthermore, the views and preferences of pregnant women can also differ, i.e., their decision-making awareness, their knowledge about the point and purpose of the research and their motivation to take part in a study.

As pointed out by Vedelø and Lomberg in their review [10], the predominant challenge for researchers conducting RCTs is to ensure that the implementation of the standardized intervention is consistent in all of the study centers, taking the different framework conditions there into account and the fact that the involved nursing staff may have no experience conducting clinical studies. Vedelø and Lomberg therefore recommend that research teams carrying out RCTs under challenging conditions should publish their experiences in dealing with challenges and hindrances to promote the implementation of future studies [10].

Midwives in German maternity hospitals frequently have little experience as investigative midwives in clinical trials. Even internationally, experience with RCTs on complex interventions in maternity hospitals is not well developed, as evidenced in an orientation search in PubMed: In the years 2010 to 2020, findings that of 61 RCTs were published in which a complex intervention was conducted in a hospital maternity ward. Of these RCTs, only 13 were conducted multicentrically in 2 to 41 hospitals with samples of 116 to 12.227 participants [11–23], 2 of which were transnational [22, 23]. In 12 articles [10–12, 14–22], methodological aspects were hardly reported. Only three of the articles [17–19] outlined the recruitment process and only Gau et al. [17] estimated that more than 50% of recruited pregnant women will drop out due to various reasons, like preterm birth, cesarean section, birth in another hospital or non-adherence of the study protocol. For this reason and with regard to supporting junior researchers, it is particularly important to share experiences conducting multicenter RCTs with the professional community, such as in maternity hospitals in Germany.

### Maternity care in German hospitals

Maternity care in Germany is characterized by centralization and insufficient personnel: in the past 20 years, the number of maternity hospitals in Germany has been reduced by 36% [24], and at the same time, the number of births has increased by 16% [25]. The workload is high for hospital midwives, and an average of 1,9 positions per hospital are vacant [26]. Obstetric care is also characterized by high intervention rates [27]: in 2019, the rate for labour induction was 22%, oxytocic drugs were administered in 25% of all births, and 31% of all pregnancies ended with a caesarean section [28]. Based on the country-specific context, this article describes the unexpected challenges that confronted the study team during the course of the clinical trial “BE-UP” despite careful planning and presents strategies for solving them that have been proven in daily practice.

### Overview on the RCT “BE-UP”

The *BE-UP trial* (acronym for *Birth environment – Upright position*) is based on four Cochrane Reviews [29–32] and a pilot study from Canada [33] and is an active controlled superiority trial with a two-arm parallel design [34]. Its aim is to increase vaginal births (VB) and, specifically, to test the effect of a redesigned birthing room (intervention) in hospitals on the probability of VB. By increasing VB, the rate of cesarean sections will be reduced, which in Germany is higher, as would be expected [35], and is associated with increased maternal and infant morbidity [36]. This trial is also in line with the *International Childbirth Initiative’s* principles of mother-friendly care [37], the Guideline by the *National Institute of Health and Care Excellence* [38] and the *International Confederation of Midwives* [39] calling for the provision of a birth environment in hospitals that fosters normal births. It is also in accordance with the Royal College of Obstetricians and Gynaecologists’ (RCOG) recommendations regarding the equipment of birth rooms [40] and the recently proclaimed 9th German national health goal “Health in Childbirth” [41].

**Fig. 1:**
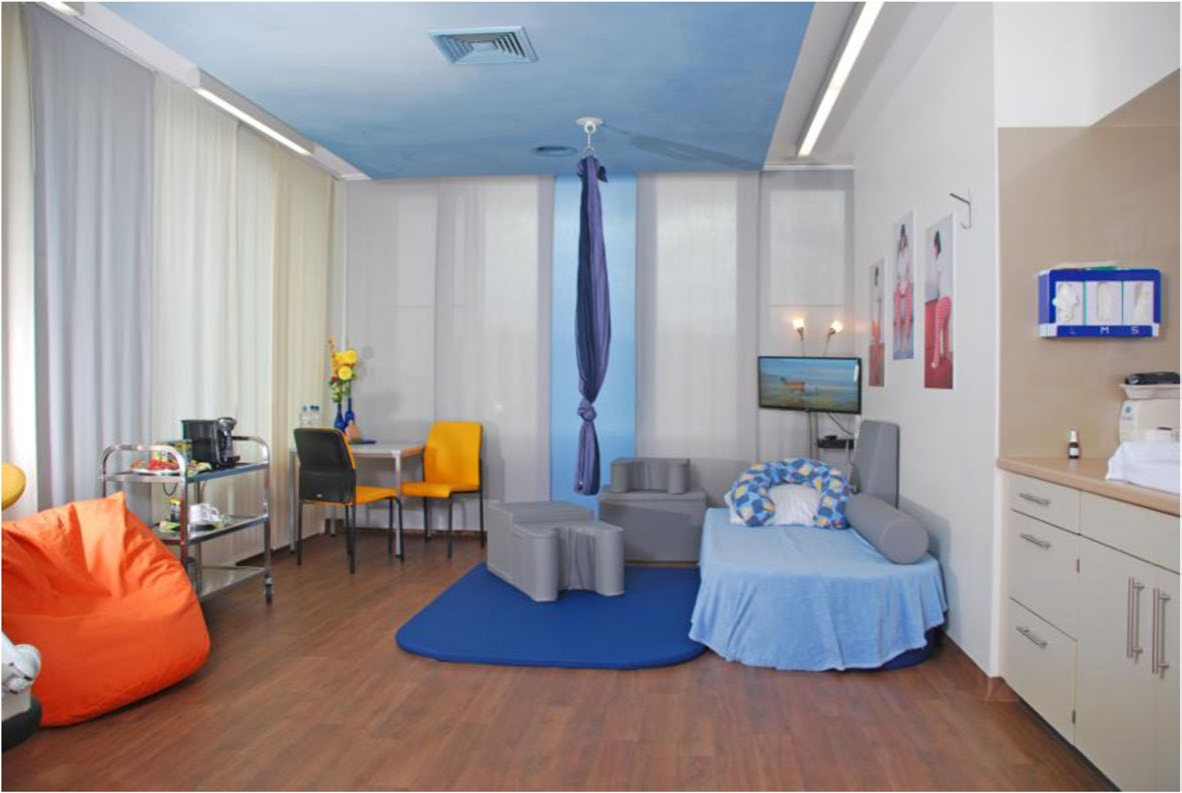
Example of the BE-UP birthing room

The complex intervention ‘redesigned birthing room’ contains specially designed features that are absent in the control birthing room. The normal birthing bed, which is otherwise in the center of the room, is either covered over or removed from the room (Fig. 1 “BE-UP birthing room at Paracelsus Hospital Henstedt-Ulzburg; permission granted by “Paracelsus Klinik Henstedt-Ulzburg”).

Instead, the setup consists of a floor mat, a 40 cm thick mattress and five foam elements (two cubes, one roll, one birthstool cushion, one back cushion). In addition, there is a beanbag, a monitor showing natural scenes, a dimmable floor lamp, a sitting area with a table and two chairs, and a snack bar with hot and cold drinks and sweet and/or savoury snacks. Each room also has three photo posters showing women in various upright postures. According to experienced midwives and various studies [42, 43], the individual components provide the birthing mother with opportunities for self-determination, relaxation and distraction so that she feels motivated to move around and stay in an upright position.

**Fig. 2:**
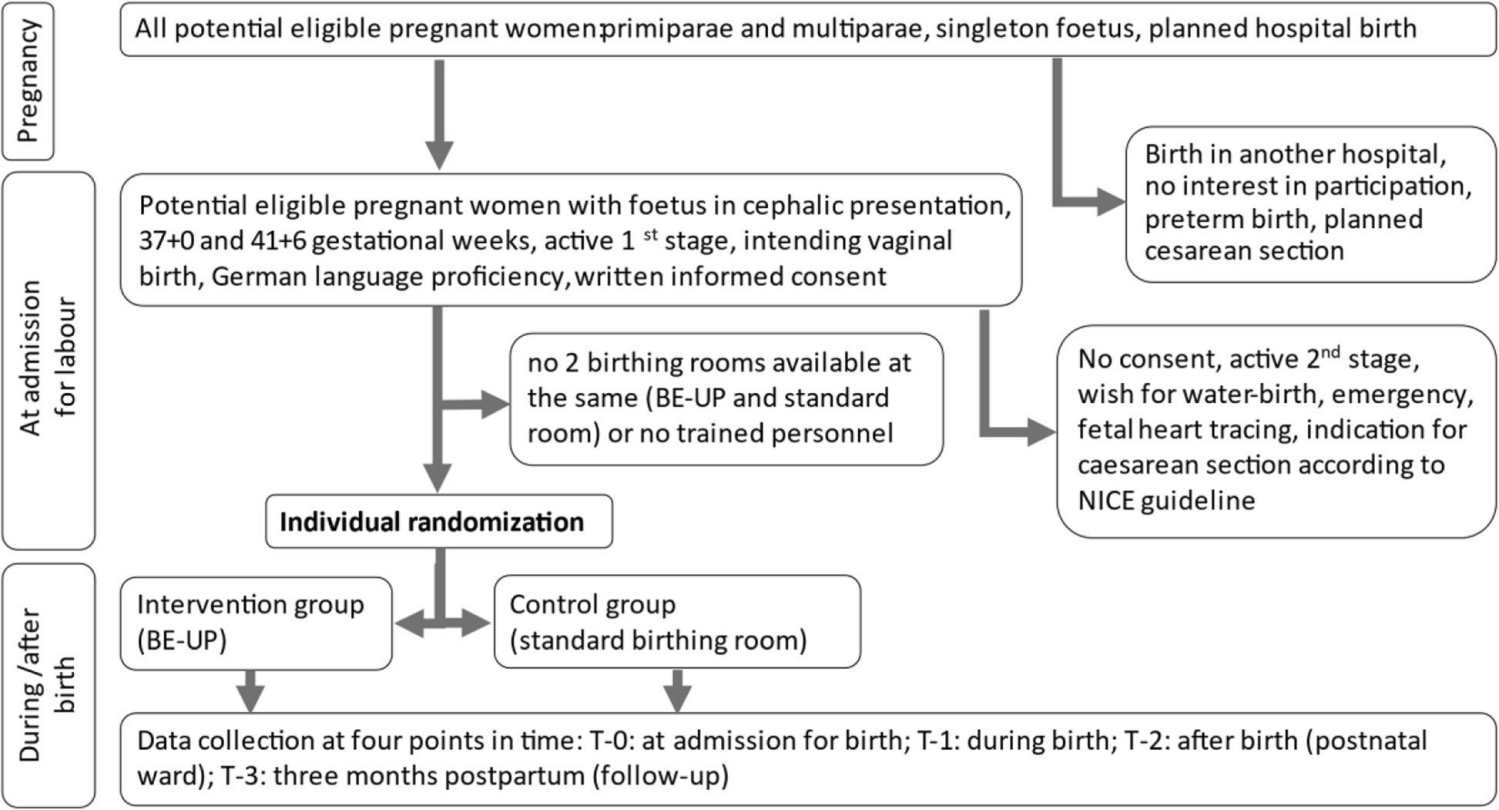
Procedure of patient enrolment

The changed setup is intended to improve the physical and emotional client-centered outcomes, a higher self-determination during birth, as well as fewer medical interventions, fewer subsequent cesarean section in future pregnancies and lower healthcare costs for interventions. The statistical calculation included a power of 90% with a 5% significance level and a dropout rate below 10% determined a sample size of 3800 women to detect a change of 5 absolute percentage points (from 74 to 79%) in the prevalence of VB (primary outcome). An increase in VB by 5% from a baseline value of 72% (421,241 VB in hospitals in Germany) to 77% would result in an additional 21,062 women per year who experience a VB instead of a cesarean section. Primiparae and multiparae who were planning their birth in a BE-UP hospital had to be informed about the study at an early stage, mainly for ethical reasons. Patient enrolment followed in a further step (see Fig. 2). At first contact and at admission for labor, staff checked to see if the inclusion criteria applied which were as follows: single fetus in cephalic presentation at term (between 37 + 0 weeks to 41 + 6 weeks pregnant), active first stage of labor and planning a VB. Pregnant women were excluded if they were in active second stage, wished for a water birth or were not able to understand the oral and written information about the trial. Women were also excluded if an evidence-based risk existed for the woman or her baby (for a detailed description see the study protocol [34]). Women who gave their written informed consent are randomized individually and centrally controlled via online application.

Data are collected at admission, during and after birth as well as at three months postpartum; data verification on site (hospitals) is done by external monitors; data management is carried out by an independent coordination center for clinical trials.

To monitor the standard implementation of the study protocol, to identify challenges and problems early on and to establish a participating relationship for constructive problem solving, visits every 2 months by members of the study team were arranged.

The BE-UP study started in 2017 in 12 maternity hospitals. Based on the total sample size calculation and the number of possible participants who might be recruited per month and participating hospital, it was decided to contact only those maternity hospitals dealing with at least 800 births per year. Recruitment started in April 2018. During the study, 6 hospitals dropped out for various reasons (change of leadership, poor recruitment of study participants, unfilled posts for midwives and physicians, building reconstruction); therefore, and with the aim of boosting the lagging recruitment rate, the number of participating hospitals was increased to 17. Additionally, the recruitment period was extended by 15 months until 31 May 2021.

### Project-related and process-oriented challenges for hospital staff

During the course of the study, besides numerous problems and issues that were rather easy to address or resolve (e.g. change of the head midwife or physician, defective bluray monitor), key challenges emerged in five sectors that are reflected as follows: 1) selection of and support for cooperating hospitals; 2) establishment of a process of requesting informed consent; 3) individual, digital real-time randomization; 4) standardized birthing room; and 5) GCP-compliant documentation. In each following section, the relevant initial conditions in the participating hospitals will be outlined before describing the challenges, their temporal occurrence and the solution strategies taken.

### Selection of and support for cooperating hospitals

In Germany, midwives are authorized to conduct physiological births. They support and care for birthing mothers independently in hospitals and cooperate with an obstetrician in pathological incidences. Midwives working in maternity hospitals generally have a high workload; nearly two-thirds of all midwives regularly look after three birthing mothers at the same time during one shift; in about a third of their shift they deal with unrelated additional work, such as cleaning, and are only seldom able to take a break; due to the poor working conditions, staff shortages prevail in many places since no applications for the vacant posts are made [26].

#### Challenges

In the planning phase of the BE-UP study, the challenge was to find hospitals where the rate of vaginal births was relatively low and thus potentially capable of improvement. Moreover, the staff had to be sufficiently motivated to want to achieve this improvement. Since at least the BE-UP birthing room plus one other normal birthing room are required to carry out individual randomization, the hospitals must at the same time have an adequate number of birthing rooms available in comparison to the number of births. Furthermore, the layout of the normal birthing rooms (as the control group), in which many hospitals in Germany provide such things as pezziballs, ceiling ropes and birthing stools, should be clearly distinguishable from the BE-UP birthing room in order for the effect of the complex intervention to be examined properly.

Considering that the study can only be successfully implemented if the whole team has developed sufficient motivation to want changes in obstetrics to take place, it was important immediately after short contact to determine whether effective cooperation existed between midwives and physicians and whether they were interested in the study. This was difficult for the study team to recognize, especially if the head physician was very impressed by the study. In such cases, the midwives might feel patronized. In addition, thought had to be given to how personnel with little experience of clinical trials should be supported in conducting the study and their motivation strengthened during the study’s 36 months duration.

#### Solutions

The goal of the study team was to first address the head midwife rather than the medical director since most of the workload would be carried out by the midwives. To obtain hard data, we requested obstetrical data from the hospital’s perinatal report.

To inform the personnel about participating in the study, staff meetings were held during which the study team presented the study and responded to questions. After the cooperation contract had been signed, several introductory events were held in each hospital to save their time resources, despite considerable expenditure of time and money for the study itself. During the course of the study, staff meetings and further training sessions were also used to instruct new staff members. Since financial resources are limited in a publicly funded study, an expense allowance of 20 Euros per study participant was planned as an incentive for the hospital, which was intended to compensate for the expenditure of one additional hour required for informing the study participants and the documentation of the additional study data; however, this compensation had little effect because only seldom was the obstetric unit’s staff allowed to have it at their own disposal. We informed the staff about new announcements concerning the study and gave them helpful tips and tricks via email and the password-protected study website. We also coordinated press announcements for the hospitals when the 100th or 250th baby was born within the BE-UP trial, all with the intention of providing continuous support and motivation to recruit women for the study.

During the course of the study, the study team organized several one-day study meetings for representatives of the staff in all of the BE-UP hospitals to facilitate interaction between them and to promote a sense of community and identification with the study. To raise participation preparedness, travel and meal expenses for two people of each hospital were reimbursed from the study’s budget. The study team also presented interesting reports in regular newsletters for the hospitals’ obstetrical teams (e.g., managing shoulder dystocia in upright maternal posture). Moreover, the participating hospitals were informed every month via email about the current recruitment situation.

### Establishing a process of requesting informed consent

In Germany, pregnant women are free to choose whichever hospital they prefer for birth, but in most gynecological practices, the women are advised to sign up in a hospital in the close vicinity. Information about the hospitals and the care they provide can be obtained from the respective website or at information events. Expectant parents often visit antenatal classes held by freelance midwives or attend parenting classes in a hospital. At that point in time and about 6 weeks before their calculated date of birth, when most of the pregnant women are registered for giving birth and a patient’s record is set up, fundamental data about the women are already available when they arrive for the actual birth. Therefore, for the first they are personally informed about the BE-UP study at registration. In contrast, there is not always sufficient time to refer to BE-UP studies during medical-diagnostic activities in special obstetrical risk consultations.

#### Challenges

At the beginning of the study, the study team realized that in the busy everyday hospital routine, the challenge for the BE-UP study centers is to draw the attention of all potential study participants – i.e., all pregnant women with a singleton pregnancy – at an early stage to the possibility of participating in the BE-UP study. Only then would the women have enough time to give their informed consent in writing. The coronavirus pandemic has complicated matters because normal information meetings and antenatal classes were cancelled for weeks and pregnant women could register for birth only over the phone.

#### Solutions

To meet the challenge, a multifaceted information strategy was planned to reach pregnant women as early as possible and to support the midwives and obstetricians as much as possible in the education about the study. Comprehensive information for potential study participants was prepared and presented on the study website. There, the study centers were listed, and explanatory materials approved by the ethics committee, i.e., information about study participation and informed consent for pregnant women, were made available for download (www.be-up-studie.de). All the text and image material was compiled with the help and participation of user representatives. With the aim of relieving the hospital staff and supporting recruitment, a multiple strategy was realized, consisting of printed materials (“quick response” [QR] code, information card, flyers, website), each with increasingly more detail. When recruitment was rather slow, the study team sent out QR code information cards and flyers to the gynecological practices and midwives in the vicinity of the hospital, asking them to pass the information on to their pregnant patients.

To help pregnant women understand the information about the BE-UP study more easily, the study team commissioned two short films, one explaining the scientific target of the BE-UP study and one showing the process of participating in the study from the perspective of the women. The films were presented on the hospitals’ websites, on the study website, and in some cases in the obstetric units’ waiting rooms. To help the maternity staff respond to occurring difficulties, various practical support measures was developed: a laminated page with the inclusion and exclusion criteria, a short text for focused consultations with the pregnant women who had not heard about the study when they arrived for the actual birth, and BE-UP stickers with the study website’s URL, which could be stuck in the pregnancy record book (Mutterpass) to remind an interested woman of the study, or else in their hospital record to indicate that she intends to participate in the BE-UP trial.

From the start of the study, the team was in close contact with the hospital IT departments until their websites had been set up satisfactorily with information about the BE-UP trial. During the course of the study, the IT departments added links on the hospital website to reach the study website, particularly the short films and special information about the situation in the coronavirus pandemic. The study team’s advice that being a BE-UP hospital would have a positive impact on its public relations image was warmly received not only at the beginning of the study. When certain milestones were achieved, for instance, the hundredth birth within the BE-UP study, the press announcements for the hospitals were adjusted appropriately, uploaded to the hospital’s websites and sent to the editors of local newspapers.

### Individual, digital real time randomization

When a pregnant woman arrives in the hospital to give birth, the first examination usually takes place in the admission room. Normally, she is then taken to a birthing room that she cannot select herself.

In the case of study participation, the inclusion and exclusion criteria were additionally checked, any remaining queries from the potential study participant were clarified, the completeness of the written informed consent was checked, and the midwife or physician confirmed with his/her signature that the woman had been adequately informed. Then, digital real-time randomization (block randomization stratified by parity) is conducted – a proven procedure provided by the University’s Coordination Center for Clinical Trials that also handles the data management of the BE-UP study. When the allocation to either the intervention group or the control group has taken place, the woman is taken to the corresponding birthing room.

#### Challenges

Already in the planning phase, a major challenge regarding the individual randomization was considered: in the BE-UP trial the intervention refers to an entire space, i.e. birthing environment (complex intervention). This resulted in special requirements: first, both the BE-UP birthing room and another normal birthing room must be vacant so that random allocation to the intervention or control group can take place. Second, when taking part in the study, the women cannot choose a room, even if two rooms are vacant at the same time. For ethical reasons, this might be perceived as being problematic because in recent years, the self-determination of a birthing woman during an actual birth has been increasingly recognized and furthered.

Since there is a limit to the number of available birthing rooms (frequently all the rooms are occupied in everyday life in a hospital), the staff has to undertake effective room management. This means that the BE-UP birthing room has to be cleaned as quickly as possible so that it is available for randomization. Moreover, for a participant to be included in the study, as many of the midwives as possible should be instructed about the study so that the study implementation can take place with the specified accuracy and fidelity, and the data collection requirements are also fully met in cases where a colleague has to take over when shifts change. Last, during the recruitment period, randomization problems due to the absence of an online connection or to operational deficiencies of the randomization software needed a precautionary solution.

#### Solutions

When the recruitment phase began, the study team advised the midwives on how best to facilitate randomization of women. We recommended the midwives of the participating hospitals to arrange for the BE-UP birthing room to be the last one occupied and to use it primarily only for the study so that as many randomizations as possible can be made. At the initial monitoring visits, the obstetric staff reported on difficulties in recruitment, also due to the large number of part-time employees who did not have an overall view of the implementation of the study. Therefore, a contact person for BE-UP was designated who was given the additional task of identifying those women admitted during the shift who could be included at that particular time.

To increase the willingness of pregnant women to take part in the study, the study team asked the staff to emphasize that nobody knew which room was better for the woman and that she had a 50% chance of giving birth in the BE-UP birthing room. It is, however, essential that the women be informed that they can assert their wishes regarding upright body posture and mobility in each birthing room and that the predefined quality standards for care in the hospital will be adhered to, signifying that disadvantages are not to be expected due to randomization.

At the beginning of the study, each of the hospitals was given an iPad for randomization. In the course of the study, it was found that there were often fewer inhibitions and difficulties if their own online devices, such as smartphones or one of the obstetric unit computers, were used. Six sealed opaque emergency randomization envelopes were additionally provided so that the staff had an alternative should problems occur during online randomization; in this way, no potential study participants would be lost.

### The standardized birthing room

Generally, birthing rooms in German maternity hospitals have a “technological” setup [44]. In the center of the room is an electrically adjustable birth bed, there is a surgical lamp, an emergency anaesthetic unit, a paediatric care unit with a heat lamp and a sink and cupboards for storing material. Quite often, there is a pezziball, a rope hanging from the ceiling and/or a birthing stool. The lights can be centrally dimmed, and if requested, the staff can provide drinks or snacks.

#### Challenges

The study team was aware of the fact that a new setup must fulfil the hospital’s hygiene standards and that staff must be able to work in the BE-UP birthing room according to the existing regulations. In each hospital, one of the birthing rooms had to be chosen as BE-UP birthing room, which meant that the standard birth bed was concealed by a paravent or removed, and that the other elements of the BE-UP concept (see above: Overview on the RCT “BE-UP”) found place. Even though the larger share of the costs for equipping the BE-UP birthing room was financed by the research sponsor, the hospitals had to cover the costs for the paravent and the snack bar.

During the initial visits in the hospitals interested to take part in the study, the particularly challenging aspects of the available birthing rooms were identified: the limited size of the rooms (very little space for a mattress, floor mat, table and chairs), the built-in power and functional cables for the birth bed and frequently insufficient room for the birth bed outside the birthing room. When the first study participants were already recruited, the study team became aware of the possibility of the foam elements slipping on the floor mat and the cool surface of the mattress. Also, after a few months of recruitment, midwives reported on backache as a cause of unusual working postures in the BE-UP birthing room. Furthermore, staff expressed concerns about emergency situations, such as shoulder dystocia.

#### Solutions

While the study was being conceptualized, the elements in the BE-UP birthing room (complex intervention) were designed in cooperation with midwives and user representatives to conform optimally to the needs of a birthing mother requiring upright posture and mobility, distraction, relaxation and self-determination. To encourage the acceptance of the changes being made when setting up the BE-UP birthing rooms in the cooperating hospitals, we adopted a strong collaborative approach. In order to boost motivation in trial implementation, and to align the BE-UP elements with the existing colour design of the birthing room, the hospital staff were able to select the colours for the floor mat, mattress, foam elements, beanbag and chairs in order to achieve a good match with the existing colour concept in the birthing room. To accommodate the cramped space, we also offered a smaller table. Thus, the BE-UP elements themselves were standardized, while the variations in color and size had no effect on the function of the individual elements.

From the start of the study, the study team endeavoured to effectively support the hospital staff: for the new way of working with upright birthing postures, the hospital was given excerpts from the e-book “The physiological birth” on the iPad as well as the new edition as a printed copy. To stop the foam elements from slipping, the team provided anti-slip material for once-only use and to make the mattress surface more pleasant, special terry-cloth sheets were provided. When the midwives in the BE-UP birthing room complained about backache, during one of the study meetings, special training in back-saving midwifery work was offered. To offer teams greater confidence in handling shoulder dystocia with the birthing woman in an upright position, we had a recognized expert develop a handout, a laminated copy of which is available in the BE-UP birthing room.

### GCP-compliant documentation

Many hospital midwives are very discontented with their jobs; the reasons are manifold – no breaks, habitual overtime and standing in for others as well as unrelated additional work [26, 45]. Above all, the high amount of documentation, which accounts for approximately 10% of the daily workload, is a particular strain on day-to-day working life; midwives are frequently only able to do the necessary documentation when their shift is over [26], and this has increased in recent years [45].

#### Challenges

In a clinical trial, the documentation of data should correspond to the guidelines of “good clinical practice” (GCP), and the respective demands are high. In obstetrical hospitals in Germany, documentation is normally digital. With the aim of avoiding double documentation on the occasion of the study, we aimed for digital documentation and direct use of routine data. However, during this preparation it became clear that various types of documentation and administration software are in use; at the same time, responsibility for documentation software rests with software providers based on legal contracts, and access to documentation software is linked to hospital-specific routines. This meant that it was probably impossible to obtain a homogeneous digital documentation.

#### Solutions

Since the study team did not want to burden the hospital staff with additional unfamiliar documentation software, case report forms (CRFs) comprising routine obstetrical data and additional trial-related items were prepared in printed form.

Simultaneously, the study team explored the possibility and willingness of the software providers to supplement the existing hospital software with a “module” for the BE-UP study to avoid double documentation and to keep the time required for the trial’s documentation as low as possible.

Upon request of the study team and within the frame of their service contract, the developers of the software systems GeDoWin®, Viewpoint® and Nexus® prepared a solution for the automatic transfer of routine data into a digital BE-UP case report form. Thus, approximately 50% of the approximately 100 items could be incorporated in the CRFs at the push of a button. In eight of the 17 participating hospitals, there were either system-specific IT hurdles or work organizational, hospital-specific hurdles; otherwise, the team opted for handwritten documentation.

Multistage monitoring was implemented to check the data quality: monitoring visits every four weeks served to check the ongoing recruitment process and to inspect and validify the data in the CRFs before sending them to the Coordination Center for Clinical Trials. The Lead Monitoring Officers, who are always easily reached by telephone, visit the hospitals every two months to check whether the complex intervention is being implemented according to the study protocol, whether recruitment is running as planned, and to deal with any special cases or challenges. Finally, they handled any enquires resulting from the plausibility checks of the Coordination Center for Clinical Trials that it undertook in the course of its query management.

## Discussion

In this article, the key challenges facing the implementation of a multicenter RCT’s complex intervention that emerged in five sectors were presented, and pragmatic solution strategies were outlined. The study team applied them a priori and during the course of the study, thus ensuring GCP-compliant implementation as well as achieving the calculated sample size, even though only when the recruitment phase had been extended.

The application of effective project management strategies is essential for an RCT to be conducted successfully; Arundel (2018) identified six key themes, namely support, communication, processes, resources, training and ethos [46]. The article at hand expands and specifies Arundel’s findings in relation to an RCT in maternity hospitals staffed by midwives and obstetricians, most of whom have little or no research experience. Future groups of researchers are thus supported when planning RCTs in maternity hospitals with similar settings. A thorough reflection on the components and their design is important in the conception of complex interventions [47] to achieve sufficient contrast to the control intervention, taking interactions during obstetrical care and contextual conditions into consideration [43, 48–50]. In addition, the opportunity for hospital staff to assert their personal preferences, such as the desired colour of various intervention components, strengthens both motivation and commitment without changing or affecting the function of the elements and thus jeopardizing standardization.

The careful selection of the study centers is of fundamentally high importance when planning an RCT [51]. In addition to objective care data such as the number of obstetric cases, heterogeneity of clients and regional location, in the field of obstetrics, the available facilities and hospital-specific care processes are highly relevant for assessing the feasibility of implementing a trial. In hospitals where no appointments are given for consultation and birth enrollment, it is difficult to impart trial information to the pregnant women early enough. A high proportion of part-time staff can be an impediment to establishing implementation routines within the team, such as the continuous appraisal of everyday situations regarding available rooms and personnel, which is a prerequisite for the continuing enrolment of study participants. Moreover, the lack of research experience and a high workload within the obstetrical team can result in not all the potential study participants receiving the required explanations about the study and therefore are not included in the trial. Staff with little research practice can profit from practical exercises and concrete phrasing suggestions, for instance, when the woman is already in labor.

The BE-UP’s multimodal information concept with increasingly detailed information for potential study participants established with the participation of the user representatives has fulfilled its purpose. Moreover, the involvement of user representatives in the entire planning and implementation process ensured that not only information content was presented in a relevant, appealing and easy-to-understand way [52], but that the elements of the BE-UP birthing room were useful and appealing from the women’s point of view. The production of several low-budget, 5-min long video films was yet another user-oriented source of information that was worth the extra cost: both of the short films were accessed approximately 300–400 times each month, confirming that even in obstetrical settings, videos are yet another way of presenting information about clinical studies [53]. This was confirmed all the more when the normal face-to-face information meetings, antenatal classes and birthing enrolments were suspended due to the COVID-19 pandemic; the numbers of users accessing the videos and the additional short audio files then increased by around a third.

In the planning stage of an RCT, the biggest threat to the randomization of study participants, the most important reason for difficulties in this direction so far was is the overestimation of the potential number of includable pregnant women in the planning stage of an RCT, resulting in a required extension of the recruitment period [8, 54]. The planned monthly recruitment figures per hospital in the BE-UP study were based on the assumption that every two days a woman could be enrolled for the BE-UP study, a cautious estimation made by international colleagues. However, despite careful overall planning, unforeseen events occurred during the implementation phase that impeded recruitment, in particular the closure of other hospitals in the vicinity and the resulting unexpected increase in births in BE-UP hospitals with the consequent persistent lack of free birthing rooms for randomization. This overloading of the systemic conditions could not be influenced by the study team. Overall, the recruitment curve in the first few months of the study grew very slowly, demonstrating that much time is needed on the part of the obstetrical teams to deal with the processes of change and adaptation. In addition, it took a longer time to motivate many of the midwives and physicians for the study. Other reasons for the slow recruitment were staff shortages and pregnant women who did not have adequate knowledge of the German language. Later, the lockdown measures of the COVID-19 pandemic impeded early access to potential study participants. During the recruitment phase, it was foreseeable that six hospitals could not increase their very low recruitment numbers so that they had to be withdrawn, and a large number of new hospitals had to be found to increase the recruitment rate. Even though this was a purposeful measure, the recruitment period had to be extended by 15 months to attain the targeted number of study participants.

As was determined in a qualitative evidence synthesis, the recruitment of study participants is also influenced by the personalities of the recruiters themselves: through their own beliefs and power, they act as gatekeepers [55]. In the BE-UP study, higher recruitment numbers were not achieved in hospitals where the midwives were willing to accept the new conditions, such as midwifery care without a birth bed. However, besides organizational difficulties and lack of time, the recruiters can experience intellectual and emotional conflict if they are confronted with a contradictory situation resulting from their clinical role and their simultaneous role as recruiters [56]. For instance, in BE-UP, the midwives might find themselves in a conflict of roles if they have to conduct an explanatory interview about the study when the mother is already in labour. Even denying a mother her wish to use the BE-UP room without participating in the study or having to hand over the woman’s care to a colleague can also be experienced as conflictual. The experiences gained by the midwives during the study as well as their courses of action are currently the subject of qualitative studies.

The practical implementation of the real-time block randomization of study participants was technically very well planned and implemented. However, the use of an online randomization service was hampered if the hospital staff had little or no experience using digital ways of communication. The additionally offered emergency randomization envelopes with concealed group allocation therefore allowed a low-threshold solution for situations in which the online connection could not be established or digital randomization did not work for other reasons.

At the beginning of the study and in the interest of cooperative partnership, the study team was eager to enable hospital staff to have a say in decision-making, for instance, when selecting colours for mattresses, foam cubes and terrycloth sheets. This kind of adaptation of the elements to the color design of the birthing room promoted the involvement and motivation of the staff without undermining the standardization of the study.

During the 2-month visits to the hospitals, the study team developed strategies for effective cooperation between themselves as researchers and the hospital staff [10] to support the obstetrical team constructively. Thus, in a large part of the BE-UP hospitals their motivation to recruit study participants was strengthened; they were increasingly enabled to cope with everyday hurdles in trial implementation and willing to work in the new standardized birthing room. This means that the strategy to develop and maintain a supportive and positive relationship with the contact persons proved effective. Due to the study team’s high social competence, efforts to convey empathy, appreciation, and commitment were convincing.

In the BE-UP trial, weekly meetings in the study centers were not possible for a total of 17 hospitals – contrary to the recommendations of other studies [57]. Instead, alternating regional and central study meetings with representatives from the participating hospitals were held, which took either half a day or a whole day and were well attended. The participants greatly appreciated the opportunity to exchange experiences among themselves.

To ensure GCP-conform documentation, the principles “collect only as much data as necessary”, “simplicity in implementation” and “best possible adaptation to existing documentation routines” were applied. The decision, to seek and establish continuing advice and support by the independent Coordination Center for Clinical Trials, which was responsible for data and query management, resulted in optimal data handling and quality. During intensive on-site monitoring, the data could be checked for completeness, correctness and readability in cooperation with the staff, and query forms from the Coordination Center for Clinical Trials filled in. Cooperation with three providers of software used in a total of 8 hospitals was helpful to the respective staff.

In retrospect it must be stated that the monetary allowance to compensate staff for the extra work in informing study participants and for the additional documentation was too low in the BE-UP study to be an effective incentive for recruitment, especially since these amounts were not available for the use of maternity staff in all hospitals.

## Conclusions

Research groups who are planning RCTs in maternity hospitals in settings with only a few midwives and physicians with research experience should carefully check the factors relating to work organization and personnel that might influence the local recruitment of study participants. In future studies, especially in such a setting, recruitment should be estimated more conservatively. When applying for an RCT and in view of the setting of the study, the principal investigator and the study team should conduct a careful and forward-looking analysis and reflection of the framework conditions of everyday maternity care to anticipate possible challenges in the practical implementation of an RCT. However, not all challenges can be anticipated in the planning of a study; therefore, the study team needs to be flexible and must react promptly to any problems that threaten recruitment or the required implementation of the complex intervention.

It is a basic requirement that the staff is convinced of the study and its purpose. Together with the staff from the participating hospitals, thought should be given to how clinic-intern processes that are relevant for conducting the study could be adapted to correspond with the study’s requirements. Understandable and practical solutions must be worked out together so that also midwives and obstetricians who have little research experience maintain their motivation to implement a complex intervention according to study protocol. This article at hand corresponds to the requirement to report on experiences in the implementation of a multicenter RCT, especially in the setting of an obstetric department. This contribution to practice-related findings provides relevant information to reflect both on concrete, quality-relevant challenges and on implementation strategies with an eye to the future to plan for adequate resources in the application for funding right from the start. Future research projects could examine in more detail the perspectives of midwives as recruiters and determine the concerns which inexperienced midwives and obstetricians might have regarding carrying out informed consent as well as how role conflicts might be eased that hamper the recruitment of study participants [58].

## Data Availability

The datasets used and/or analyzed during the current study available from the corresponding author on reasonable request.
